# Ruptured peripheral superior cerebellar artery dissecting aneurysms associated with primitive trigeminal artery: a case report

**DOI:** 10.1186/s12883-022-02741-y

**Published:** 2022-06-06

**Authors:** Sayaka Ito, Kazushi Higuchi

**Affiliations:** Department of Neurosurgery, Japanese Red Cross Nagahama Hospital, Miyamae-cho 14-7, Nagahama, Shiga Japan

**Keywords:** Collateral circulation, Endovascular intervention, Flow alteration, Subarachnoid hemorrhage, Treatment strategy

## Abstract

**Background:**

Persistent primitive trigeminal artery (PPTA) is a remnant of the carotid-vertebrobasilar anastomoses in the embryo. Although PPTAs are discovered incidentally in most cases, altered hemodynamics may lead to increased risk of stroke. To the best of our knowledge, no reports of PPTA associated with superior cerebellar artery (SCA) dissecting aneurysms have been published in the English language.

We describe the case of a patient who presented with subarachnoid hemorrhage (SAH) due to ruptured peripheral SCA dissecting aneurysms in association with PPTA. Additionally, we discuss the relationship between PPTA and peripheral SCA aneurysms and the treatment of peripheral SCA aneurysms.

**Case presentation:**

A 43-year-old woman presented with acute onset of headache and nausea and admitted to our hospital. She was diagnosed with SAH due to ruptured left SCA dissecting aneurysm(s) and had undergone digital subtraction angiography. The left vertebral angiography showed aneurysmal dilatations of the left S2 segment (lateral pontomesencephalic segment) along with dissection through the segments of S2 and S3 (cerebellomesencephalic segment). It also showed ipsilateral PPTA. The left vertebral artery (VA) had normal caliber and the basilar artery segment proximal to the orifice of the left PPTA was not hypoplastic. The patient underwent proximal parent artery occlusion at the S2 segment via the left VA and was successfully treated with no neurological deficits having lasted 5 months.

**Conclusions:**

The flow alteration with PPTA may have influenced the formation of SCA dissection in this patient. Further studies are needed to understand the etiology and treatment outcomes of peripheral SCA aneurysms better.

## Background

Persistent primitive trigeminal artery (PPTA) is a remnant of the carotid-vertebrobasilar anastomoses in the embryo, which is a connection of primitive carotid artery and paired bilateral longitudinal neural arteries supplying the developing fetal brain. PPTA is present in 0.1–1.0% of individuals [1]. Although PPTAs are discovered incidentally in most cases, altered hemodynamics may lead to an increased risk of stroke [2–4]. To the best of our knowledge, there are no reports of PPTA associated with superior cerebellar artery (SCA) dissecting aneurysms published in English language.

Herein, we describe a patient who presented with subarachnoid hemorrhage (SAH) due to ruptured peripheral SCA dissecting aneurysms in association with PPTA. The dissecting lesion involved two SCA segments and was treated with endovascular embolization of the proximal parent vessel and ruptured aneurysms.

Our literature review identified only three similar cases [5, 6]. Our report emphasizes the significance of recognizing this rare condition. In addition, we discuss the relationship between PPTA and peripheral SCA aneurysms as well as the treatment of peripheral SCA aneurysms. In this report, we used the nomenclature for SCA segments suggested by Rodriguez-Hernandez [7] (Table 1).

**Table 1:** Nomenclature for SCA segments

S1	Anterior pontomesencephalic segment
S2	Lateral pontomesencephalic segment
S3	Cerebellomesencephalic segment
S4	Cortical segment

## Case presentation

**Fig. 1:**
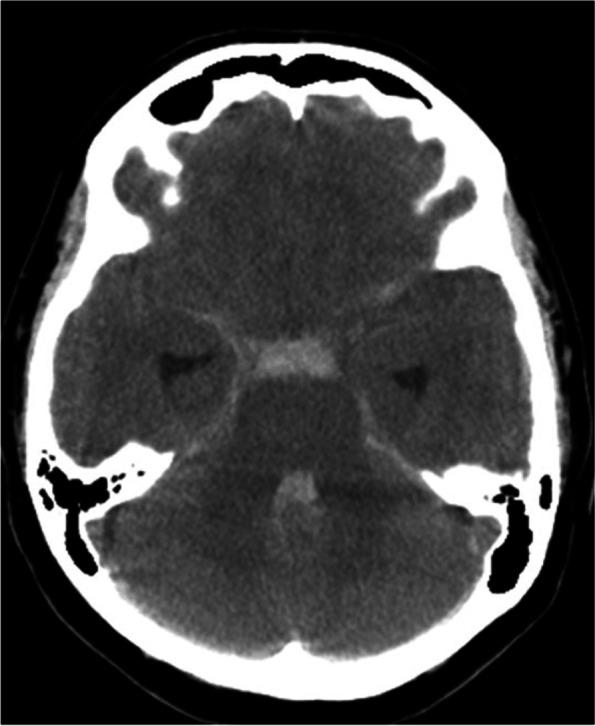
Computed Tomography scan of the patient at admission. Computed Tomography showing subarachnoid hemorrhage

**Fig. 2:**
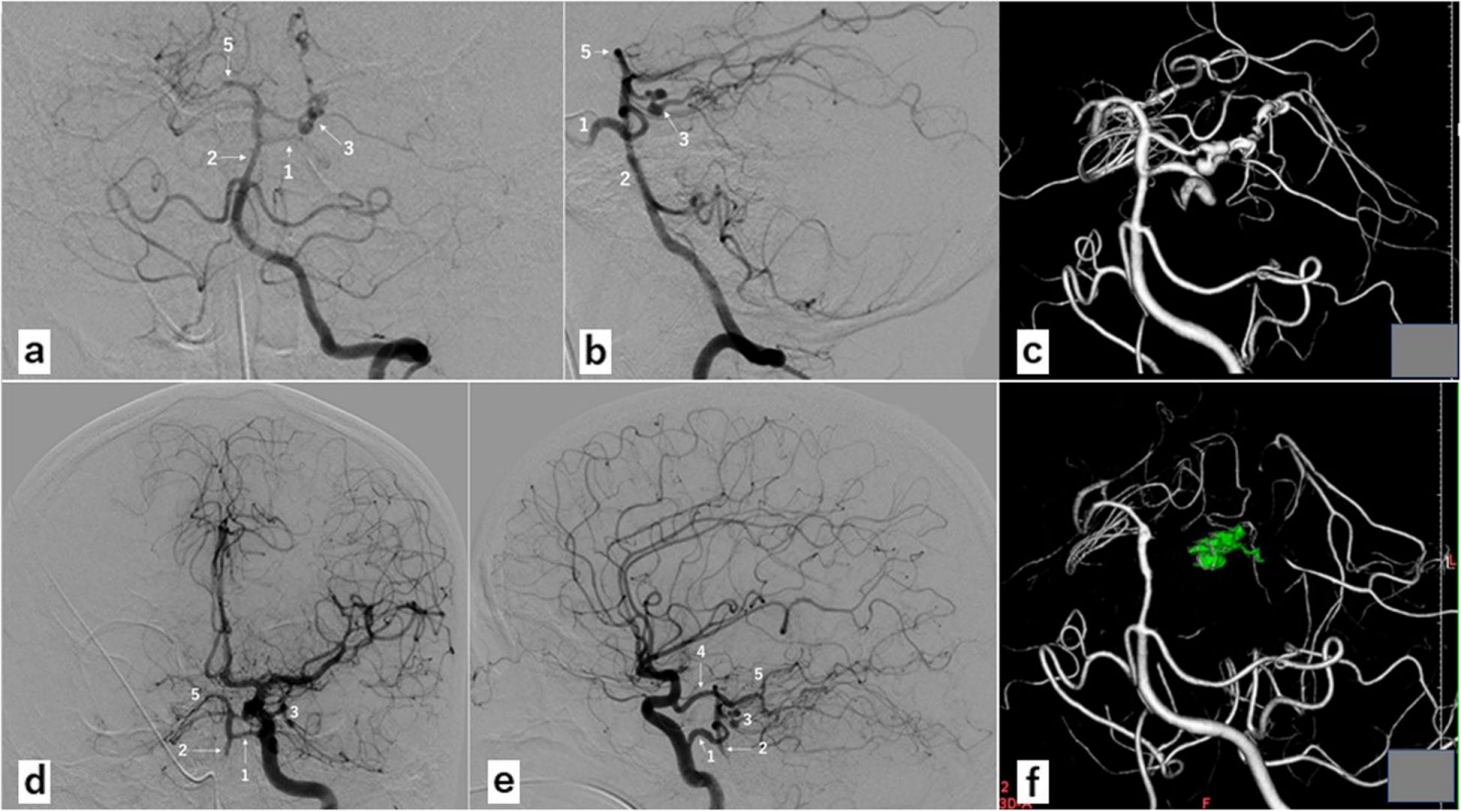
Digital subtraction angiography of the patient before (**a-e**) and after (**f**) the endovascular intervention

A 43-year-old woman with no significant medical history was brought to our hospital by ambulance because of acute onset of headache and nausea. On admission, she complained of severe headache without any other neurological deficits. Her brain CT/CT angiography revealed SAH (Fig. 1) and left SCA dissection with aneurysms. She was diagnosed with Hunt & Kosnik grade 2 SAH due to ruptured left SCA dissecting aneurysm(s). Subsequently, she underwent digital subtraction angiography under general anesthesia for further evaluation and definitive treatment. The left vertebral angiography (VAG) showed apparent aneurysmal dilatations of the left S2 along with dissection through the S2 and S3 segments. The S2 segment was extremely tortuous with two aneurysms. Although the rupture point is usually located on the aneurysms, it could not be identified. The dissection involved the entire S2 segment and rostral trunk of S3 segment. The left vermian branch and caudal trunk of the S3 segment were intact. The left VAG showed ipsilateral PPTA with to-and-fro blood flow pattern between the basilar artery (BA) and left internal carotid artery (ICA). The left PPTA joined the BA proximal to the orifices of bilateral SCAs and distal to the orifices of bilateral anterior inferior cerebellar arteries (AICAs) (Fig. 2a-c). The left vertebral artery (VA) had a normal caliber, whereas the right VA was hypoplastic. The BA segment proximal to the orifice of the left PPTA was not hypoplastic. The left P1 was not identified on any angiography. The left ICA angiography (ICAG) showed the ipsilateral anterior cerebral artery and middle cerebral artery as well as the ipsilateral PPTA supplying the BA distal and proximal to the orifice (Fig. 2d, e). The left posterior communicating artery supplied blood to the left posterior cerebral artery (PCA) distal to P1. The right PCA was visualized by the left VAG and bilateral ICAGs. Based on the aforementioned findings, the left S2 aneurysms were considered to be accessible via the left VA during endovascular intervention. The balloon test occlusion covering the orifices of bilateral SCAs showed good collateral crossflow to the healthy left S4 and vermian branch from the left AICA and posterior inferior cerebellar artery (PICA). The distal part of the dissection was located at S3, which was not the rupture point, and was inaccessible because of the proximal extreme tortuosity at S2. The planned strategy was proximal parent artery occlusion (PAO) at S2, including occlusion of ruptured aneurysms, without bypass. The left SCA was cannulated with an Excelsior SL-10 microcatheter (Stryker Neurovascular, Tokyo, Japan) over a manually curved Traxcess (Micro Vention, CA, U.S.A.). The microcatheter was advanced beyond the distal aneurysm. Nine metallic coils were sequentially deployed in a distal-to-proximal order. At the end of the procedure, the proximal left SCA was patent and the SCA distal to S1 showed complete obliteration on angiography from BA distal to AICA. The left VAG showed that the retrograde arterial flow of the left vermian branch and rostral and caudal trunks of S4 were supplied by the left AICA and PICA without visualization of the dissecting S2 and S3 (Fig. 2f). There were no perioperative complications. Magnetic resonance imaging/angiography revealed no infarctions. Her hospital course was good. She was discharged home at the 21st day of hospitalization with modified Rankin Scale of 0. At the 4-month follow-up, a diagnostic angiogram revealed complete obliteration of the dissected portion of the SCA and significant collateral crossflow to SCA distal to the dissected portion. There was no rebleeding or recanalization of the dissected vessel at 5 months.

The left vertebral angiography (VAG) before the treatment showing ipsilateral persistent primitive trigeminal artery (PPTA) (1 in a and b), basilar artery (BA) (2 in a and b), aneurysmal dilatations of the left S2 segment (3 in a and b), and right posterior cerebral artery (PCA) (5 in a and b). The dissection involving the entire S2 segment and the rostral trunk of the S3 segment of superior cerebellar artery (SCA) with the left vermian branch and caudal trunk of the S3 segment intact (c). The left PPTA joining the BA proximal to the orifices of bilateral SCAs and distal to the orifices of bilateral anterior inferior cerebellar arteries. The left vertebral artery had a normal caliber. The BA segment proximal to the orifice of the left PPTA was not hypoplastic.

The left ICA angiography showing ipsilateral PPTA (1 in d and e), BA (2 in d and e), aneurysmal dilatations of the left S2 segment (3 in d and e), left posterior communicating artery (4 in e), and left right PCA (5 in d and e). The PPTA supplying the BA distal and proximal to the orifice.

The left VAG after the treatment showing the retrograde arterial flow of the left vermian branch and rostral and caudal trunks of S4 being supplied by the left AICA and PICA without visualization of the dissecting S2 and S3 (f).

## Discussion and conclusions

### Relationship between PPTA and peripheral SCA aneurysm

PPTA is one of the remnant arteries of the carotid–vertebrobasilar anastomoses in the embryo [1, 4]. The blood is supplied from the ICA to the primitive vertebrobasilar systems [8]. PPTA was first described in a cadaveric brain by Richard Quinn in 1844 and was angiographically reported in alive patients by David Sutton in 1950. Thereafter, with the development of neuroimaging modalities and accumulation of cadaveric studies, the incidence of PPTA was elucidated to be 0.1–1.0% [1]. Persistent carotid–vertebrobasilar anastomoses are usually subclinical [1, 4].

PPTAs have been found incidentally with other brain lesions, including ruptured or unruptured aneurysms [1, 8], carotid-cavernous fistula [9], ischemic stroke [1, 10–12], arteriovenous malformation [1, 13], cavernous malformation [1], moyamoya disease [1, 3], and brain tumors [1]. PPTA-related symptoms occur due to the irritation of cranial nerves III-VI because of compression of the outer wall of the cavernous sinus [1].

We thoroughly searched major electronic databases for studies on PPTA and SCA aneurysms. The present case is the first in the published English literature related to PPTA associated with peripheral SCA aneurysm(s).

In our case, the PPTA showed Saltzman combined type 1 + 2, in which the ipsilateral side was type 2 and contralateral side was type 1 [14]. The anastomosis supplied the bilateral SCA as well as contralateral PCA. The BA proximal to the insertion of the PPTA was not hypoplastic and the left VA was dominant, through which bilateral SCAs received as much blood supply as from PPTA. Based on the findings above, flow alteration due to PPTA may have affected the flow dynamic stress to the left SCA as well as upper BA, right SCA, and right PCA. The stress may have been one of the triggers of SCA dissection. In such a situation, other affected vessels must be carefully observed.

### Treatment of ruptured peripheral SCA aneurysm

SCA aneurysms account for 1–5% of all intracranial aneurysms [15]. In general, the SCA aneurysms can be divided into two types: BA-SCA junction aneurysms and peripheral SCA aneurysms (i.e., aneurysms other than those located at the BA-SCA junction). Peripheral SCA aneurysms are distinct from the other SCA aneurysms because of their anatomic location and higher incidence of fusiform or dissecting morphology, which complicates their treatment [16]. Although some studies have reported good results of microsurgery for the management of SCA aneurysms [15], endovascular treatment is widely performed because it is less invasive and as safe as microsurgery, especially for peripheral SCA aneurysms [6, 16, 17].

There is good collateral circulation among the SCAs, AICAs, and PICAs through the vermian arcade, paramendian branches and perforators of the basilar artery, which prevent infarction after occlusion [18]. However, SCA occlusion produces a distinctive clinical picture due to infarction of the cerebellum, dentate nucleus, superior cerebellar peduncle, and long sensory pathways in the tegmentum of the rostral pons [19]. Although ischemic stroke is a potential complication of SCA occlusion, collateral blood flow may limit ischemic infarction and the outcome is usually good [2]. Nevertheless, the treatment-related complications depend on the occluded SCA segment. The definitive treatment of peripheral SCA aneurysms is controversial [6, 16], which is probably because the disease is rare and the lesions vary in terms of location, length, and involvement of branches/perforators.

Our patient had a dissecting artery at the left S2 and S3 segments. SCA dissection involving two or more segments is extremely rare. A thorough review of the published English literature revealed only three reported cases, which were treated by endovascular intervention [5, 6].

Alurkar et al. reported a case of ruptured S2 to S4 dissection treated with PAO at S2 and S3, followed by a good outcome without any recurrence or rebleeding. The authors stated that minimally invasive endovascular PAO may be a good treatment option for these aneurysms because SCA occlusion is well tolerated in the presence of good collaterals [5].

Ascanio et al. reported two cases of ruptured dissecting SCA aneurysms, including a case of S1 and S2 dissection, and a case of S2 and S3 dissection. The lesions were successfully occluded at the distal part of the dissection to spare S1 without rebleeding. In the latter case, the dissection extended to the S1 and S4 segments of the SCA at the 2-month follow-up. The authors concluded that although PAO at the distal part of the dissecting section may be reliable for preventing acute rebleeding, long-term observation was required [6].

Our case involved dissection at the left S2 and S3 segments with aneurysm rupture at S2. We treated our patient with PAO at S2 after confirming the retrograde crossflow. Fortunately, the lesion was not at S1, from which the perforators to the midbrain arise; therefore, the risk of brainstem infarction was not very high. However, extension of the dissection proximal and distal to the treated site may occur, as described previously. Therefore, long term observation is required for our patient with PPTA-derived altered flow in SCAs and PCA.

We reported a case of ruptured peripheral S2 and S3 dissecting aneurysm associated with PPTA, which was treated with PAO. This was the first case with PPTA and associated SCA dissecting aneurysm. The flow alteration with PPTA may have influenced the formation of SCA dissection in our patient. The determination of the optimal treatment strategy for peripheral SCA aneurysm is difficult because it is a rare entity with a variety of lesions. Further studies are needed to better understand the etiology and treatment outcomes of peripheral SCA aneurysms.

## Data Availability

The data that support the findings of this case are available on reasonable request from the corresponding author. The data are not publicly available due to privacy or ethical restrictions.

## Abbreviations

PPTA: Persistent primitive trigeminal artery
SCA: Superior cerebellar artery
SAH: Subarachnoid hemorrhage
VAG: Vertebral angiography
BA: Basilar artery
ICA: Internal carotid artery
AICA: Anterior inferior cerebellar arteries
VA: Vertebral artery
ICAG: ICA angiography
PCA: Posterior cerebral artery
PICA: Posterior inferior cerebellar artery
PAO: Proximal parent artery occlusion
